# An activating mutation in AGEF-1, a putative Arf GEF, causes yolk extrusion from *C. elegans* embryos

**DOI:** 10.17912/micropub.biology.002060

**Published:** 2026-06-17

**Authors:** Clare FitzPatrick, Olga Skorobogata, Ali M. Fazlollahi, Kimberley D. Gauthier, Christian E. Rocheleau

**Affiliations:** 1 Department of Anatomy and Cell Biology, McGill University, Montreal, QC, CA; 2 Metabolic Disorders and Complications Program, RI-MUHC, Montreal, QC, CA; 3 Division of Endocrinology and Metabolism, Department of Medicine, McGill University, Montreal, QC, CA

## Abstract

*C. elegans* AGEF-1 , an ortholog of human ARFGEF1 and ARFGEF2, functions with ARF-1 , ARF-5 and the AP-1 clathrin adaptor to regulate membrane trafficking. Similar phenotypes induced by the *agef-1 ( vh4 [E1028K])* allele and *agef-1 (RNAi)* suggested that *agef-1 ( vh4 )* was a hypomorph. Here we report that *agef-1 ( vh4 )* results in extrusion of yolk from the embryo. This is suppressed by RNAi of *agef-1 , arf-1 , arf-5* but not AP-1. Based on structure of the yeast AGEF-1 ortholog, Sec7p, the E1028K change is predicted to activate AGEF-1 . Thus, Arf GTPase cycling is likely required to regulate trafficking with AP-1 but not with Arf effectors regulating yolk trafficking.

**Figure 1. The yolk blob phenotype of agef-1(vh4) requires the ARF-1 and ARF-5 GTPases but not the AP-1 complex:** **(A)** Alignment of amino acids from the HDS2 domains of *C. elegans* AGEF-1 and *S. cerevisiae* Sec7p. The activating L1376D mutation in Sec7p corresponds to L1029 of AGEF-1 and is adjacent to E1028 which is changed to a Lysine in the *agef-1 ( vh4 )* mutant. Identical residues are shown in dark purple and similar residues in light purple. **(B)** Differential interference contrast (DIC), epifluorescence and merged images of an *agef-1 ( vh4 )* three-fold stage embryo expressing the VIT-2 ::GFP transgene *bIs1* . Scale bar, 5μm. **(C)** Merged DIC and epifluorescence images of wild-type (WT) and *agef-1 ( vh4 )* three-fold stage embryos and an L1 larva (upper right) expressing VIT-2 ::GFP and treated for RNAi targeting *agef-1* , *arf-1* , *arf-5* , *apg-1* , *apm-1* , *aps-1* and an empty vector (EV) control. VIT-2 ::GFP is localized to the intestine in wild type but is consolidated in droplets or blobs in *agef-1 ( vh4 )* that pool between the embryo and the eggshell as well as internally as can be seen in the hatched L1 larva (upper right). Scale bar, 10μm. **(D)** Most embryos have VIT-2 positive blobs that both accumulate internally and are extruded from the embryo **. (E)** RNAi targeting *agef-1* , *arf-1* and *arf-5* suppressed the *agef-1 ( vh4 )* yolk blob phenotype but did not reduce the fluorescence intensity of VIT-2 ::GFP in bean-stage embryos **(F)** . **(G)** RNAi targeting *apg-1* , *apm-1* or *aps-1* did not suppress the *agef-1 ( vh4 )* yolk blob phenotype. **(H)** Model that the activating mutation in AGEF-1 (star) increases the levels of active GTP-bound ARF-1 and ARF-5 (green) that can engage effectors. Since GTPase cycling is important for AP-1 mediated vesicle trafficking the net result is an inhibition of AP-1 trafficking events (red) during vulva induction and regulating lysosome size in coelomocytes. In the case of embryonic yolk trafficking Arf GTPase cycling does not appear to be required to activate an unknown effector (?; green) to misdirect yolk. An unpaired t test was used to determine significance of percentages or fluorescence intensity. ns, not significant, * P<0.05, **P<0.01, ***P<0.001, ****P<0.0001. Number of embryos quantified were 45 (D), 102-151 (E), 29-66 (F), and 95-121 (G) per condition.

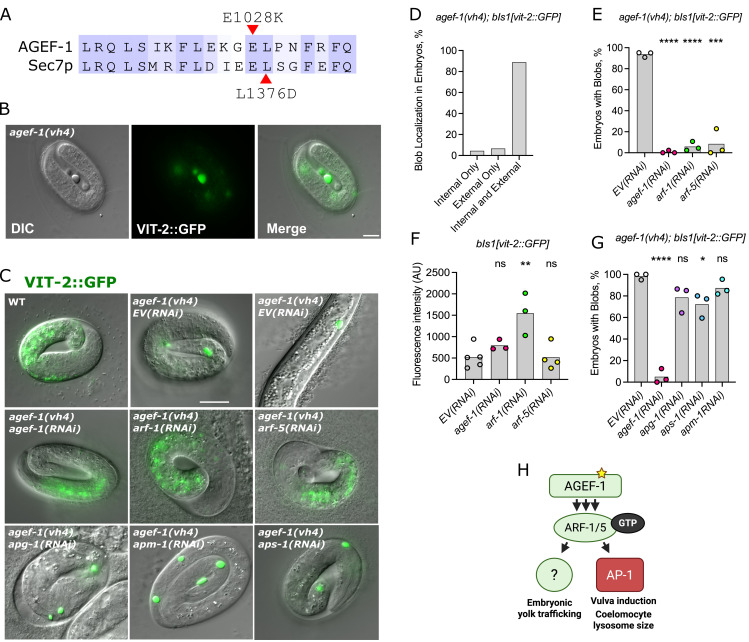



## Description

*C. elegans* yolk is comprised of lipid associated with six apoB-like vitellogenins. Yolk is synthesized in the hermaphrodite intestine and secreted into the pseudocoelom (body cavity) before being endocytosed into maturing oocytes (Kimble and Sharrock, 1983; Grant and Hirsh, 1999; Hall *et al.* , 1999; Perez and Lehner, 2019). During embryogenesis yolk granules becomes distributed amongst the dividing blastomeres. Once the primordial intestine is formed, yolk accumulates in the intestinal cells (Bossinger and Schierenberg, 1996). Little is known about how yolk is distributed during embryogenesis.

Arf GTPases are regulators of membrane trafficking that cycle between a GTP-bound “on” state and a GDP-bound “off” state regulated by guanine nucleotide exchange factors (GEFs) and GTPase activating proteins (GAPs), respectively (Jackson and Bouvet, 2014). When bound to GTP, Arfs can interact with effector proteins to regulate membrane trafficking. *C. elegans* AGEF-1 is a putative GEF orthologous to yeast Sec7p and human ARFGEF1 and ARFGEF2 that activate class I and II Arf GTPases (Togawa *et al.* , 1999; Sato *et al.* , 2006; Ishizaki *et al.* , 2008; Skorobogata *et al.* , 2014). We previously reported that a missense allele, *agef-1 ( vh4 )* , that results in a Glutamic acid to Lysine change in the HDS2 domain ( Figure 1A ) caused mislocalization of the LET-23 /Epidermal Growth Factor Receptor (EGFR) in the vulva precursor cells and caused enlargement of endosomes and lysosomes in coelomocytes (Skorobogata *et al.* , 2014). Both phenotypes were phenocopied by *agef-1 (RNAi)* , and a deletion allele was zygotic lethal, suggesting that *agef-1 ( vh4 )* was a hypomorphic allele (Tang *et al.* , 2012; Skorobogata *et al.* , 2014).

Here we report that *agef-1 ( vh4 )* mutant embryos had a yolk trafficking phenotype. Unlike wild type, *agef-1 ( vh4 )* embryos accumulated extraembryonic yolk in between the eggshell and the developing embryo as determined by differential interference contrast microscopy and confirmed with a yolk protein fusion VIT-2 ::GFP ( *bIs1 )* ( Figure 1B ). In nearly all *agef-1 ( vh4 )* embryos VIT-2 ::GFP was found in pools or blobs rather than in the intestine ( Figure 1B, C). While many yolk blobs were clearly floating between the embryo and the eggshell, many appeared to accumulate internally ( Figure 1D ). Analysis of newly hatched larvae confirmed that 53% (n=41) had yolk blobs that failed to extrude from the embryo ( Figure 1C, upper right). However, it was not clear if this phenotype was caused by loss of *agef-1* or a background mutation as we did not observe a yolk blob phenotype by *agef-1 (RNAi)* (0% yolk blobs across 3 replicates, *n=* 72-84 embryos/replicate).

Recent structural analysis of the yeast ortholog of AGEF-1 , Sec7p, revealed that the HDS2 domain interacts with the SEC7 GEF domain, blocking the interaction with Arf1 (Brownfield *et al.* , 2024). Consistent with this being an autoinhibitory interaction, an engineered L1376D mutation in the HDS2 domain greatly increased Sec7p GEF activity toward Arf1 *in vitro* . This Leucine is conserved in AGEF-1 and is adjacent to the Glutamic Acid that is mutated in *agef-1 ( vh4 )* suggesting that this allele could also be an activating allele ( Figure 1A ). To test if *agef-1 ( vh4 )* is indeed an activating allele, we performed *agef-1* RNAi on the *agef-1 ( vh4 )* mutant and found that it potently suppressed the yolk blob phenotype while control empty vector (EV) RNAi had no effect ( Figure 1C, E). Thus, *agef-1 ( vh4 )* is likely a hypermorphic allele and the yolk blobs may be a result of increased Arf GTPase activity.

AGEF-1 functions with ARF-1 and ARF-5 to antagonize LET-23 /EGFR localization and signaling during vulva development (Skorobogata *et al.* , 2014). We found that RNAi of either *arf-1* or *arf-5* strongly suppressed the *agef-1 ( vh4 )* yolk blob phenotype suggesting that both are required ( Figure 1C, E). To ensure that this suppression is not caused by decreased yolk uptake into maturing oocytes we measured fluorescence intensity of VIT-2 ::GFP in bean-stage embryos treated with RNAi targeting *agef-1 , arf-1* or *arf-5* . We found no decrease in VIT-2 ::GFP fluorescence intensity from these RNAi knockdowns and in fact *arf-1 (RNAi)* significantly increased the levels of VIT-2 ::GFP ( Figure 1F ). Thus, ARF-1 and ARF-5 activity are required for the yolk blob phenotype of *agef-1 ( vh4 )* without blocking yolk uptake into embryos.

The *agef-1 ( vh4 )* mutant behaved as an activating allele in the context of yolk trafficking but acted as a loss of function mutation during LET-23 /EGFR-mediated vulva development and in regulation of lysosome size in coelomocytes (Skorobogata *et al.* , 2014). In mammalian cells Arf1 activation is required for AP-1 recruitment (Stamnes and Rothman, 1993), but GTP hydrolysis appears to be required for AP-1 uncoating and subsequent vesicle trafficking (Tanigawa *et al.* , 1993; Zhu *et al.* , 1998; Meyer *et al.* , 2005). We hypothesized that the AP-1 complex would not be required for the *agef-1 ( vh4 )* yolk blob phenotype. We found that RNAi targeting AP-1 complex components *apg-1* , *apm-1* or *aps-1* did not suppress the *agef-1 ( vh4 )* yolk blob phenotype despite causing a potent dead egg phenotype ( Figure 1C, G). Therefore, the aberrant yolk trafficking seen in *agef-1 ( vh4 )* happens independently of the AP-1 clathrin adaptor complex.

Here we show that *agef-1 ( vh4 )* is likely a hypermorphic allele and that aberrant AGEF-1 activity results in mislocalization of yolk during embryogenesis. Unlike other *agef-1 ( vh4 )* phenotypes that are phenocopied by RNAi the yolk trafficking phenotype is suppressed by *agef-1 (RNAi)* as well as with RNAi targeting *arf-1* and *arf-5 .* However, the *agef-1 ( vh4 )* yolk trafficking phenotype is not suppressed by RNAi targeting components of the AP-1 complex. These data are consistent with a model whereby increased AGEF-1 activity activates ARF-1 /5, which in the case of yolk trafficking, engages an unidentified effector that does not require cycling like AP-1 ( Figure 1H ). The strong phenotypes caused by RNAi targeting either *arf-1* or *arf-5* could suggest a requirement for both GTPases. However, we noted that *arf-1* and *arf-5* coding sequences and hence their RNAi clones share stretches of identical nucleotide sequences that could cause some cross reactivity. Therefore, further experiments with mutants will be required to determine whether one or both GTPases regulate yolk trafficking. Furthermore, we used VIT-2 ::GFP as a proxy for yolk, thus we cannot exclude the possibility that yolk comprised of other vitellogenins are trafficked normally in *agef-1 ( vh4 )* mutants.

A similar yolk trafficking phenotype was reported for loss *alfa-1 /C9orf72* and *smcr-8 /SMCR8* , where ALFA-1 and SMCR-8 were found to regulate endolysosomal trafficking (Corrionero and Horvitz, 2018). Intriguingly, structural and biochemical data suggests that C9orf72 and SMCR8 function together as an Arf GAP (Su *et al.* , 2020; Su *et al.* , 2021). If this is the case, then Arf GTPase activity would be increased in *alfa-1* and *smcr-8* mutants. Further analysis will be required to determine if ALFA-1 and SMCR-8 function in a common pathway with AGEF-1 to regulate yolk trafficking.

Human ARFGEF1 variants are associated with developmental delay with and without epilepsy (Takata *et al.* , 2019; Thomas *et al.* , 2021; Xu *et al.* , 2022). While most ARFGEF1 mutants introduce premature stop codons and frameshifts some are missense mutations. Notably the I1180R mutation in the HDS2 domain of ARFGEF1 could be an activating allele as the analogous residue in yeast makes contact with the Sec7 GEF domain in the autoinhibited state (Brownfield *et al.* , 2024). If so, we would expect the corresponding mutant in *agef-1* would cause a yolk trafficking phenotype. Thus, *C. elegans* embryonic yolk trafficking could be used to assay the effects of conserved ARFGEF1 disease alleles.

## Methods

Wormbase ( https://wormbase.org ) was an invaluable resource to the planning and execution of this work (Sternberg *et al.* , 2024). All strains were maintained at 20°C on Nematode Growth Medium (NGM) and fed HB101 *E. coli* as a food source, as previously described (Brenner, 1974; Stiernagle, 2006). All strains were derived from the wild type N2 strain. RNAi by feeding was performed as previously reported (Kamath *et al.* , 2003). All RNAi experiments were performed a minimum of 3 times. All images were collected on an Axio Imager A1 using the Axiocam 305 with Axio Vision software (Zeiss). Embryos were mounted on 2% agarose pads in water and imaged using glass coverslips between 0.16 to 0.19 mm (Fisher). For yolk blob scoring, a minimum of one VIT-2 ::GFP positive blob was the threshold for the presence of a yolk blob. A percent of blobs present was taken after each round of RNAi. For total VIT-2 ::GFP amounts, images were captured as previously described (Chotard *et al.* , 2010b) and mean fluorescence intensity was measured using the selection tool brush in Fiji, and subtracting background ( https://imagej.net/software/fiji/ ). Statistics and graphical analysis were performed using Graph Pad Prism 10. Sequence alignment was performed using EMBOSS Water Pairwise Sequence Alignment (PSA) ( https://www.ebi.ac.uk/jdispatcher/psa/emboss_water?format=clustal ). Visualization and final alignment was performed using Jalview version 2.11.4.1 ( https://www.jalview.org/ ).

## Reagents

**Table:** 

**Strain**	**Genotype**	**Source**
QR14	*bIs1 [Pvit-2:: VIT-2 ::GFP; rol-6 ( su1006 )]* X	(Chotard et al., 2010); Derived from DH1033 (Grant & Hirsh, 1999)
QR201	*agef-1 ( vh4 )* I *; bIs1 [Pvit-2:: VIT-2 ::GFP; rol-6 ( su1006 )]* X	(Skorobogata et al., 2014)

**&nbsp;**

**Table:** 

**RNAi clone**	**Gene**	**Source**
L4440	Empty vector control	(Timmons & Fire, 1998)
I-2M02	*apm-1*	(Fraser et al., 2000)
I-6L22	*agef-1*	(Fraser et al., 2000)
I-7A02	*apg-1*	(Fraser et al., 2000)
III-3A13	*arf-1*	(Kamath et al., 2003)
IV-4E13	*arf-5*	(Kamath et al., 2003)
V-4F01	*aps-1*	(Kamath et al., 2003)

**&nbsp;**
